# Video Urodynamic Predictors of Outcomes After Urethral Sphincter Botulinum Toxin A Injection in Spinal Cord-Injured Patients with Detrusor Sphincter Dyssynergia

**DOI:** 10.3390/toxins17080412

**Published:** 2025-08-15

**Authors:** Cheng-Ling Lee, Hann-Chorng Kuo

**Affiliations:** Department of Urology, Hualien Tzu Chi Hospital, Buddhist Tzu Chi Medical Foundation, Buddhist Tzu Chi University, Hualien 970004, Taiwan; leecl@hotmail.com

**Keywords:** voiding dysfunction, urethral sphincter, spinal cord injury, neurogenic bladder

## Abstract

**Purpose:** Detrusor sphincter dyssynergia (DSD), a common lower urinary tract condition in patients with suprasacral spinal cord injury (SCI), can lead to urological complications and reduced quality of life. Urethral sphincter botulinum toxin A (BoNT-A) injection has been used to promote spontaneous voiding, albeit with limited success. This study aimed to identify predictive factors for treatment success. **Methods:** This retrospective analysis included 207 patients (157 males and 50 females) with chronic SCI and varying DSD grades treated with urethral sphincter BoNT-A injection. Each received 100 U of onabotulinumtoxinA via transurethral sphincter injection. The primary outcome was voiding efficiency (VE) and symptom improvement, assessed via global response evaluation 3 months post-treatment. Baseline videourodynamic parameters were used to predict success. **Results:** Successful outcomes were observed in 33.8% of patients. These patients were older and had higher voiding pressure, maximum flow rate (Qmax), voided volume, bladder contractility index, and VE, as well as lower post-void residual (PVR) volume and bladder outlet obstruction index. Patients with SCI and DSD grade 1 had the highest success rate (65.7%) compared to those with DSD grade 2 (14.3%) or 3 (7.1%). Patients with DSD grade 3 had the highest failure rate (55.8%). Multivariate analysis showed that higher Qmax and lower PVR significantly predicted success, consistent with lower DSD grades. **Conclusion:** Grade 1 DSD, higher Qmax, and lower PVR were associated with higher success after urethral BoNT-A injection, whereas grade 3 DSD predicted failure. Thus, careful patient selection is essential for effective DSD treatment with urethral BoNT-A injection.

## 1. Introduction

Spinal cord injury is a debilitating condition that severely impairs quality of life, causes voiding dysfunction, and potentially endangers upper urinary tract function if not properly managed [1]. In patients with suprasacral cord injury, DSD is common, leading to high voiding pressure, incomplete bladder emptying, and hydronephrosis [2]. Urethral sphincter injection of 100 U of BoNT-A has been shown to reduce urethral sphincter hypertonicity and reduce detrusor pressure during the voiding reflex [3]. Patients responding to urethral BoNT-A injections show substantial reductions in maximal detrusor pressure and maximal urethral closure pressure, along with improved quality of life [4]. However, not all patients with chronic SCI benefit from this treatment.

DSD can be classified into three grades based on urethral sphincter activity during the voiding phase [2,5]. Patients with grade 1 DSD typically urinate spontaneously or with stimulation, without high voiding pressure or large PVR volume. Patients with grade 2 DSD show intermittent urethral sphincter activity during voiding, resulting in higher voiding pressure and larger PVR compared with patients exhibiting DSD grade 1 [5]. Patients with grade 3 DSD are marked by non-relaxing or increased urethral sphincter activity during voiding, causing extremely high voiding pressure and large PVR [6]. In patients with chronic SCI and grade 2 or 3 DSD, intravesical pressure and detrusor contraction duration gradually increase, bladder compliance decreases, and the upper urinary tract may deteriorate due to vesicoureteral reflux or obstructive uropathy [7].

Urethral sphincter BoNT-A injection has been used to reduce urethral sphincter hypertonicity since 1988 [8]. After BoNT-A treatment, voiding pressure decreases, urination becomes easier, and PVR is reduced [3,4]. However, patients may be troubled by increased urinary incontinence and persistent incomplete bladder emptying [9]. For those accustomed to emptying the bladder via CIC, these side effects can be unpleasant, and patients often decline further injections once the BoNT-A effects diminish [10]. Conversely, patients with NDO and DSD may find voiding easier and require less frequent CIC when treated with combined detrusor and urethral BoNT-A injections [11]. Few studies have examined predictive factors for satisfactory outcomes of urethral sphincter BoNT-A injection in patients with chronic SCI and varying DSD grades. This retrospective study analyzed treatment outcomes, adverse events, and subsequent bladder management in patients with chronic SCI and DSD. Predictive factors for satisfactory outcomes following urethral BoNT-A injection were also evaluated.

## 2. Results

Among the 207 patients (157 males and 50 females), 78, 65, 29, and 35 had cervical, thoracic, lumbar, and sacral SCI, respectively. VUDS parameters are summarized in Table 1. DSD of any grade was present in 98.4%, 86.2%, 65.5%, and 82.9% of cervical, thoracic, lumbar, and sacral SCI cases, respectively. AD was observed exclusively in 79.5% and 35.4% of patients with cervical and thoracic SCI, respectively. Patients with cervical SCI had the highest Pdet, BOOI, and BCI but the lowest CBC and PVR. Baseline VE was similar across SCI subgroups, as was the rate of successful treatment outcomes. Overall, 33.8% of patients achieved a successful outcome, 41.1% showed improvement, and 25.1% experienced treatment failure. Baseline urodynamic parameters by DSD grade are shown in the Supplemental Table S1. Qmax, VE, and BCI were highest in grade 1 DSD and lowest in grade 3; PVR and BOOI were highest in grade 3 DSD and lowest in grade 1.

Table 2 presents clinical demographics and VUDS parameters by treatment outcome following urethral BoNT-A injection. Patients with successful outcomes were older and had higher Pdet, Qmax, voided volume, BCI, and VE, along with lower PVR and BOOI. Across all patients, those with SCI and DSD grade 1 had the highest success rate (65.7%) compared with DSD grade 2 (14.3%) and grade 3 (7.1%). Grade 3 DSD was associated with the highest treatment failure rate (55.8%). The therapeutic duration lasted for 3 to 6 months in patients with a successful or improved treatment outcome (mean 5.1 ± 0.5 months). Patients who had treatment failure after initial BoNT-A injection did not receive a second treatment in less than 6 months.

Bladder management, postoperative adverse events, and subsequent surgery for lower urinary tract dysfunction following urethral sphincter BoNT-A injection in patients with different treatment outcomes are shown in Table 3. In total, 59.4% of patients resumed spontaneous voiding, whereas 38.2% required CIC or CISC for complete bladder emptying, including 23 (11%) who could urinate after BoNT-A treatment. Postinjection, AD was reduced in severity in 75 (36.2%) patients, while 10 (5%) had exacerbated AD; dysuria persisted in 27.1% of patients, whereas urinary incontinence worsened in 16.9%. Ten patients (4.8%) underwent augmentation enterocystoplasty owing to small bladder capacity and urological complications, and three patients (1.5%) required bladder outlet surgery to enable spontaneous voiding.

Logistic regression analysis identified higher Qmax, smaller voided volume, lower PVR, greater VE, and higher BCI as predictive of successful outcomes following urethral BoNT-A injection for DSD-related voiding dysfunction. Multivariate analysis showed that higher Qmax and lower PVR were significant predictors (Table 4). These predictors correspond with lower DSD grades in patients with chronic SCI.

## 3. Discussion

This study showed that 33.8% of patients with chronic SCI and DSD achieved a successful outcome, 41.1% experienced improvement, and 25.1% failed to benefit from urethral sphincter BoNT-A injection. Patients with grade 1 DSD had the highest success rate (65.7%), whereas those with grade 3 DSD had the highest failure rate (55.8%). Postinjection, 27.1% of patients reported persistent dysuria and 16.9% experienced exacerbated urinary incontinence. Multivariate analysis identified higher Qmax and lower PVR as significant predictors of a favorable outcome.

DSD is a common lower urinary tract dysfunction in patients with suprasacral SCI [12]. Higher SCI levels and complete injuries are typically associated with more severe DSD and carry a high risk of urological complications [13]. The condition contributes to elevated detrusor pressure, chronic bladder inflammation, and reduced bladder compliance, increasing the risk of upper urinary tract damage and recurrent UTIs [6]. Patients with DSD and high PVR should be managed with CIC or CISC and require regular urinary tract follow-up [5]. In cases involving reduced bladder capacity or lacking vesicoureteral reflux/obstructive uropathy, surgical options such as augmentation enterocystoplasty or bladder outlet surgery may be necessary to lower intravesical pressure and facilitate voiding through reflex, triggered, or abdominal straining methods [9].

With the advent of BoNT-A therapy for lower urinary tract dysfunction, urethral sphincter injection was initially considered a promising option to lower outlet resistance and promote spontaneous voiding in neurogenic and non-neurogenic populations [3,8,9,14]. Pediatric patients with dysfunctional voiding or DSD can also benefit from intrasphincter BoNT-A injections without voiding symptom recurrence [15].

However, despite early encouraging results, subsequent clinical trials have shown more modest success rates [10,16,17,18]. Patients with chronic SCI and DSD usually have both storage and voiding symptoms. Intradetrusor BoNT-A injection has been well documented to increase the CBC and reduce Pdet and improve urinary incontinence [19]. The urodynamic improvement of patients with spinal cord lesion and high risk of bladder augmentation may have a chance to continue BoNT-A injections without proceeding to a surgical procedure for preventing upper urinary tract deterioration [20]. Although patients with DSD and unable to void spontaneously might expect to urinate spontaneously with the aid of a urethral sphincter BoNT-A injection [21]. Patients with voiding dysfunction due to detrusor underactivity, dysfunctional voiding, or NDO with DSD may still require CIC or CISC due to incomplete emptying [10]. Additionally, 48.5% of patients with detrusor underactivity and poor compliance or NDO with DSD may experience overflow or urgency incontinence after urethral sphincter BoNT-A injection [22]. Consequently, long-term adherence to this therapy is often limited and lacks strong evidence to support the clinical application of this treatment in treating DSD [9,23]. Patients with suboptimal outcomes frequently progress to further treatments, including detrusor BoNT-A injection, augmentation enterocystoplasty, or bladder outlet procedures, such as transurethral incision of the bladder neck, transurethral resection of the prostate, or external sphincterotomy, for more definitive management [9].

The outcomes of this retrospective cohort are consistent with previously published data [9,10,22]. Treatment success did not differ significantly across SCI levels. Patients with successful outcomes were typically older and displayed higher Pdet, Qmax, voided volume, BCI, and VE, along with lower PVR and BOOI. In multivariate analysis, higher Qmax and lower PVR remained significant predictors, aligning with lower DSD grade in chronic SCI. Notably, grade 3 DSD had the highest failure rate, whereas grade 1 had the highest success rate.

The mechanism underlying the urethral sphincter BoNT-A injection is pharmacologically sound. BoNT-A injection blocks acetylcholine release at efferent nerve terminals, leading to focal muscle paralysis [24]. Following elucidation of this mechanism, BoNT-A has been applied extensively to treat DSD in chronic SCI [8,14,18,25]. However, the optimal dosing for various DSD grades remains unclear. A 100 U dose of onabotulinumtoxinA has historically been used as the standard for neurogenic and non-neurogenic voiding dysfunction [8,26,27]; however, therapeutic outcomes have been inconsistent and potentially overestimated [21]. Whether the 100 U onabotulinumtoxinA dosage is adequate for more severe DSD (grade 2 or 3) remains unresolved.

In the present study, successful outcomes were associated with older age, as well as higher Pdet, Qmax, voided volume, BCI, and VE, and lower PVR and BOOI. Grade 1 and 3 had the highest success (65.7%) and failure (55.8%) rates, respectively. These findings indicate that lower urethral resistance in patients with chronic SCI and DSD is a key predictor of treatment success. This aligns with previous research indicating that poor urethral BoNT-A treatment outcomes are associated with bladder neck dyssynergia and absent detrusor contractility [28]. However, the standard dose of 100 U onabotulinumtoxinA may be inadequate for high-grade DSD, given that BoNT-A efficacy is dose-dependent, as shown in treatments for overactive bladder and NDO [29,30]. Therefore, to improve outcomes, higher doses may be necessary in patients with severe DSD.

Treatment success did not significantly differ by SCI level. A previous study had found that 47 out of 90 (52%) patients with DSD and detrusor contraction had a successful result; However, only 1 in 9 (11%) with DSD coexisting with detrusor underactivity had limited VE and a successful outcome after urethral BoNT-A injection [28]. This could explain why 34.3% of patients with grade 1 DSD did not achieve successful treatment outcomes. Detrusor underactivity can result from neurogenic bladder wall inflammation or DSD’s inhibitory effects on detrusor function [31,32]. Currently, no effective therapy exists to enhance VE in this context; CIC remains the primary option for achieving bladder emptying postinjection [1,33].

Despite administering BoNT-A treatment, 27.1% of patients continued to report dysuria, and 16.9% experienced worsening incontinence. Although spontaneous voiding was restored in 59.4% of patients, 6.3% required intermittent CIC/CISC, 31.9% remained fully dependent on CIC/CISC, and 2.4% continued using indwelling catheters. Repeat injections were needed to maintain the therapeutic effect in patients with successful outcomes. Such limitations often lead patients to pursue surgical interventions aimed at increasing bladder capacity or lowering outlet resistance. Overall, long-term satisfaction with urethral BoNT-A injection remains modest.

Several limitations of this study must be acknowledged. First, this was a retrospective study excluding non-ambulatory patients. Second, treatment outcomes were based on subjective reports without the support of validated questionnaires or follow-up urodynamic studies. Nevertheless, the study’s strength lies in its large cohort and inclusion of all DSD grades in chronic SCI. The identified predictors for treatment success offer valuable guidance in selecting appropriate candidates for BoNT-A injection treatment.

## 4. Conclusions

In summary, this study demonstrated a 33.8% success rate in patients with chronic SCI and DSD injected with 100 U of urethral BoNT-A. Patients with grade 1 DSD showed the highest success rate, whereas those with grade 3 had the highest failure rate. Persistent dysuria and exacerbated urinary incontinence are the main adverse events after treatment. Higher Qmax and lower PVR were significant predictors of a successful treatment outcome. Collectively, these findings show that careful patient selection is essential for optimizing the therapeutic effect of urethral BoNT-A injection in the studied population.

## 5. Materials and Methods

In total, 207 consecutive patients with chronic SCI and various DSD grades during the voiding phase, treated with urethral sphincter BoNT-A injection to promote spontaneous voiding from 2002 to 2024, were reviewed. All patients had received medication to relax urethral sphincter hyperactivity or non-relaxing sphincters but failed to resume spontaneous voiding. All underwent VUDS, confirming detrusor contractility and DSD or non-relaxing urethral sphincter activity during voiding. Patients with detrusor underactivity or coordinated urethral sphincter activity during voiding were excluded. The study was conducted according to the Declaration of Helsinki, and the protocol was approved by the Ethics Committee of Hualien Tzu Chi Hospital, Buddhist Tzu Chi Medical Foundation (IRB114-124-B). Informed consent was waived owing to the retrospective nature of the study.

The VUDS procedure was detailed in our previous studies [6,9,10]. Briefly, a 6 Fr dual-channel urethral catheter was used for bladder infusion and intravesical pressure (Pves) monitoring. A rectal balloon catheter with 30 mL of saline was inserted into the rectum to measure Pabd. Pdet was calculated by subtracting Pabd from Pves. Urethral sphincter EMG was continuously recorded using surface patch electrodes placed in the perianal area. Patients with quadriplegia were investigated in the supine position, whereas those with paraplegia were evaluated in the sitting position (females) or standing position (males able to ambulate). Bladder infusion rate was maintained at 20–30 mL/min, and a C-arm fluoroscope was used to monitor the bladder and bladder outlet images during the storage and voiding phases of VUDS.

DSD grade and urodynamic terms were defined according to International Continence Society recommendations [34]. VUDS parameters included Qmax, voided volume, PVR, CBC, and VE, as well as calculated BOOI and BCI [35]. AD was defined and recorded if patients developed hypertension, bradycardia, extremity hyperreflexia, or hyperemic change above the SCI level during VUDS, at full bladder, or on spontaneous voiding [36]. DSD was graded from 0 to 3 based on urethral sphincter EMG activity during urination and urethral sphincter imaging during the voiding phase [2,37]. BoNT-A injection was administered under light intravenous general anesthesia. In total, 100 U of BoNT-A was delivered via transurethral sphincter injection, following our previously established method [26]. One vial of 100 U onabotulinumtoxinA was diluted with 5 mL of normal saline. In male patients, urethral sphincter injections were performed via the transurethral route using an injection cystoscope and a 23-gauge needle (22 Fr, Richard Wolf, and Knittlingen, Germany) with 5 injections (1.0 mL of each) circumferentially distributed in the urethral sphincter at the 2, 4, 6, 8, and 10 o’clock positions. In females, transcutaneous injections were administered to the urethral sphincter along the urethral lumen at the 2, 5, 7, 10, and 12 o’clock positions around the urethral meatus. Following the injections, a Foley catheter was placed overnight and removed the next morning. Self-voiding status was recorded during outpatient follow-up. A 3-day antibiotic course was prescribed to prevent urinary tract infections (UTIs), and medications for reducing urethral resistance were discontinued after injection. Based on our prior experience, BoNT-A’s effect on the urethral sphincter began around 3 days postinjection and peaked 2 weeks postinjection [10]. If voiding difficulty gradually developed or the PVR increased after BoNT-A injections, repeat BoNT-A treatment was carried out at least 6 months after the prior injection to avoid the antibody effect on the therapeutic efficacy of BoNT-A.

The primary outcome was VE at 3 months after BoNT-A treatment, evaluated via global response assessment from patient chart reviews over 3 months, graded from 0 to 3. Patients requiring indwelling urethral catheterization (IDC), suprapubic cystostomy, or CIC, or those with VE < 33% were classified as treatment failures (grade 0). Those with a VE of 33.3–66.7% who could urinate spontaneously or with abdominal straining were considered mildly improved (grade 1). A VE of 66.7–90.0% indicated moderate improvement (grade 2), whereas a VE of 90.0–100% indicated marked improvement (grade 3) [38]. Patients achieving grade 2 or 3 were considered to have successful outcomes. For VE recording, patients were asked to void at a strong or urgent desire during uroflowmetry. If the voided volume plus PVR was <250 mL, patients were requested to void again. Adverse effects after BoNT-A injections were also recorded. Baseline VUDS parameters were analyzed to identify predictive factors for successful treatment outcomes.

Categorical variables were presented as frequencies (proportions), whereas continuous variables were expressed as means with standard deviations. Analysis of variance was used to determine subgroup differences. The Chi-square test was employed to analyze categorical variables. To determine predictors of successful outcomes, multivariate analysis was conducted using a forward selection method. All statistical analyses were performed using SPSS for Windows (v16.0; SPSS, Chicago, IL, USA), with a significance threshold of *p* < 0.05.

## Figures and Tables

**Table 1:** Baseline demographics and urodynamic characteristics of patients with spinal cord injury stratified by injury level.

	Cervical (n = 78)	Thoracic (n = 65)	Lumbar (n = 29)	Sacral (n = 35)	Total (n = 207)	*p*-Value
Age (year)	51.8 ± 16.9	40.9 ± 14.8	42.9 ± 18.1	34.9 ± 23.4	44.3 ± 18.7	0.000
Male: Female	69:9	50:15	16:13	22:13	157:50	0.001
Pdet (cm H_2_O)	37.1 ± 22.5	30.2 ± 25.9	12.7 ± 13.9	16.9 ± 17.7	28.1 ± 23.7	0.000
Qmax (mL/s)	5.2 ± 5.9	4.9 ± 6.5	7.6 ± 9.3	3.6 ± 4.9	5.2 ± 6.6	0.109
Volume (mL)	76.7 ± 92.4	66.9 ± 97.7	109.2 ± 133.9	76.2 ± 108.7	78.1 ± 103.6	0.334
PVR (mL)	195.7 ± 157.7	264.5 ± 196.5	315.5 ± 146.8	259.6 ± 238.4	244.9 ± 188.1	0.015
CBC (mL)	272.4 ± 149.5	331.4 ± 172.5	424.8 ± 155.4	335.7 ± 216.4	322.9 ± 176.3	0.001
VE	0.31 ± 0.31	0.24 ± 0.3	0.24 ± 0.24	0.25 ± 0.34	0.27 ± 0.3	0.492
BOOI	26.7 ± 23.8	20.3 ± 28.9	−2.5 ± 19.4	9.7 ± 19.8	17.7 ± 26.3	0.000
BCI	62.9 ± 39.3	54.9 ± 41.7	50.7 ± 52.3	34.9 ± 31.1	53.9 ± 41.8	0.010
AD	62 (79.5%)	23 (35.4%)	0	0	85 (41.1%)	0.000
DSD I	33 (42.3%)	14 (21.5%)	9 (31%)	13 (37.1%)	69 (33.3%)	0.003
DSD II	21 (26.9%)	21 (32.3%)	7 (24.1%)	16 (45.7%)	65 (31.4%)	
DSD III	22 (28.2%)	21 (32.3%)	3 (10.3%)	0	46 (22.2%)	
Non-DSD	2 (1.56%)	9 (13.8%)	10 (34.5%)	6 (17.1%)	27 (13.0%)	
Successful	27 (34.6%)	23 (35.4%)	9 (31%)	11 (31.4%)	70 (33.8%)	0.340
Improved	34 (43.6%)	21 (32.3%)	11 (37.9%)	19 (54.3%)	85 (41.1%)	
Failed	17 (21.8%)	21 (32.3%)	9 (31%)	5 (14.3%)	52 (25.1%)	

**Table 2:** Baseline demographics and urodynamic parameters in relation to treatment outcomes.

	Successful (n = 70)	Improved (n = 85)	Failed (n = 52)	Total (n = 207)	*p*-Value
Age (year)	49.7 ± 17.1	42.7 ± 20.7	39.7 ± 15.6	44.3 ± 18.7	0.008
Male: female	49:21	68:17	40:12	157:50	0.343
Pdet (cm H_2_O)	31.4 ± 23.2	29.4 ± 25.3	21.5 ± 20.6	28.1 ± 23.7	0.061
Qmax (mL/s)	9.7 ± 7.7	3.8 ± 4.6	1.3 ± 3.3	5.2 ± 6.6	0.000
Volume (mL)	139.7 ± 120.5	64.7 ± 87	17.2 ± 43.7	78.1 ± 103.6	0.000
PVR (mL)	150.7 ± 119.3	246.3 ± 192.3	369.3 ± 186.5	244.9 ± 188.1	0.000
CBC (mL)	290.4 ± 158.5	310.9 ± 175.9	386.5 ± 186.6	322.9 ± 176.3	0.008
VE	0.47 ± 0.29	0.24 ± 0.29	0.055 ± 0.13	0.27 ± 0.3	0.000
BOOI	12 ± 29.2	21.7 ± 25.1	18.96 ± 22.9	17.7 ± 26.3	0.065
BCI	79.8 ± 42.9	48.5 ± 37.6	27.9 ± 23.8	53.9 ± 41.8	0.000
AD (baseline)	25 (35.7%)	35 (41.2%)	25 (48.1%)	85 (41.1%)	0.390
DSD I	46 (65.7%)	17 (20%)	6 (11.5%)	69 (33.3%)	0.000
DSD II	10 (14.3%)	46 (54.1%)	9 (17.3%)	65 (31.4%)	
DSD III	5 (7.1%)	12 (14.1%)	29 (55.8%)	46 (22.2%)	
Non-DSD	9 (12.9%)	10 (11.8%)	8 (15.4%)	27 (13.0%)	

**Table 3:** Post-treatment bladder management, adverse events, and subsequent surgical interventions after urethral sphincter BoNT-A injection in patients with different treatment outcomes.

	Successful (n = 70)	Improved (n = 85)	Failed (n = 52)	Total (n = 207)	*p*-Value
Bladder management					
SV	66 (94.3%)	51 (60%)	6 (11.5%)	123 (59.4%)	0.000
CISC	0	5 (5.9%)	0	5 (2.4%)	
CIC	1 (1.4%)	17 (20%)	43 (82.7%)	61 (29.5%)	
IDC	0	3 (3.5%)	0	3 (1.4%)	
Cystostomy	0	2 (2.4%)	0	2 (1%)	
SV + CIC	3 (4.3%)	5 (5.9%)	3 (5.8%)	11 (5.3%)	
SV + CISC	0	2 (2.4%)	0	2 (1%)	
Postoperative adverse events					
AD	1 (1.4%)	1 (1.2%)	6 (11.5%)	8 (3.9%)	0.000
Dysuria	3 (4.3%)	36 (42.4%)	16 (30.8%)	55 (26.6%)	
UTI	2 (2.9%)	3 (3.5%)	4 (7.7%)	9 (4.3%)	
UUI	16 (22.9%)	15 (17.6%)	4 (7.7%)	35 (16.9%)	
AD + UTI	0	0	2 (3.8%)	2 (1%)	
Dysuria + UTI	0	1 (1.2%)	0	1 (0.5%)	
Subsequent surgery					
None	69 (98.6%)	77 (90.6%)	48 (92.3%)	194 (93.7%)	0.218
AE	1 (1.4%)	7 (8.2%)	2 (3.8%)	10 (4.8%)	
TUI-BN	0	1 (1.2%)	1 (1.9%)	2 (1%)	
TUI-P	0	0	1 (1.9%)	1 (0.5%)	

**Table 4:** Predictive factors associated with successful treatment outcomes following urethral sphincter BoNT-A injection.

Univariate				Multivariate		
	**Odd Ratio**	**95% CI**	***p***	**Odd Ratio**	**95% CI**	***p***
Pdet (cm H_2_O)	0.988	0.977–1.000	0.057			
Qmax (mL/s)	0.823	0.772–0.878	0.000	0.870	0.776–0.976	0.018
Volume (mL)	0.990	0.987–0.994	0.000	0.999	0.993–1.006	0.861
PVR (mL)	1.006	1.003–1.008	0.000	1.004	1.000–1.008	0.026
CBC	1.002	1.000–1.003	0.050			
VE	0.032	0.010–0.098	0.000	1.114	0.093–13.292	0.932
BOOI	1.010	0.999–1.022	0.075			
BCI	0.973	0.965–0.982	0.000	0.997	0.982–1.012	0.729

## Data Availability

Data are available upon request from the corresponding authors.

## Supplementary Materials

The following supporting information can be downloaded at: https://www.mdpi.com/article/10.3390/toxins17080412/s1, Table S1: Baseline demographics and urodynamic parameters in patients with different DSD grades.

## Abbreviations

AD: autonomic dysreflexia; AE: augmentation enterocystoplasty; BCI: bladder contractility index; BoNT-A: botulinum toxin A; BOOI: bladder outlet obstruction index; CBC: cystometric bladder capacity; CIC: clean intermittent catheterization; CISC: clean intermittent self-catheterization; DSD: detrusor sphincter dyssynergia; EMG: electromyography; IDC: indwelling urethral catheterization; NDO: neurogenic detrusor overactivity; Pabd: intra-abdominal pressure; Pdet: detrusor pressure; PVR: maximum flow rate; Qmax: maximum flow rate; SCI: spinal cord injury; SV: spontaneous voiding; TUI-BN: transurethral incision of bladder neck; TUI-P: transurethral incision of prostate; UTI: urinary tract infections; UUI: urgency urinary incontinence; VE: voiding efficiency; VUDS: videourodynamic study
