# Supplementary material for: Video Urodynamic Predictors of Outcomes After Urethral Sphincter Botulinum Toxin A Injection in Spinal Cord-Injured Patients with Detrusor Sphincter Dyssynergia
Source: Toxins (Basel). 2025 Aug 15;17(8):412. doi: 10.3390/toxins17080412 (PMC12389972; doi:10.3390/toxins17080412)
Supplement: Supplementary file 1 [file toxins-17-00412-s001.zip › toxins-3768370-supplementary.pdf]

Supplementary Materials: Video Urodynamic Predictors of Outcomes after Urethral Sphincter Botulinum Toxin A Injection in Spinal Cord Injured Patients with Detrusor Sphincter Dyssynergia

Cheng-Ling Lee and Hann-Chorng Kuo *

Department of Urology, Hualien Tzu Chi Hospital, Buddhist Tzu Chi Medical Foundation, Buddhist Tzu Chi University, Hualien 970004, Taiwan; leecl@hotmail.com
* Correspondence: hck@tzuchi.com.tw

Supplemental Table 1. Baseline demographics and urodynamic parameters in patients with different DSD grades.

	DSD Ⅰ (n=69)	DSD Ⅱ (n=65)	DSD Ⅲ (n=46)	Non-DSD (n=27)	Total (n=207)	P-value
Age	54.4±15.9	36.1±18.8	43.6±14.8	39.5±19.8	44.3±18.7	<0.001
Male : Female	48 : 21	47 : 18	40 : 6	22 : 5	157 : 50	0.139
Pdet	30.1±19.9	32.4±27.8	29.0±23.7	11±13.1	28.1±23.7	0.001
Qmax	9.1±6.8	4.4±5.4	1.5±5.9	3.3±4.4	5.2±6.6	<0.001
Volume	125.0±92.4	73.1±110.9	12.6±43.3	81.9±124.9	78.1±103.6	<0.001
PVR	148.7±110.1	249.5±213.9	356.6±178.1	289.3±179.6	244.9±188.1	<0.001
CBC	273.6±156.7	322.6±197.5	369.2±175.6	371.2±142	323.0±176.3	0.013
VE	0.46±0.25	0.26±0.31	0.034±0.1	0.23±0.31	0.27±0.30	<0.001
BOOI	12.0±24.8	23.6±26.2	25.9±28.9	4.52±15.5	17.7±26.3	<0.001
BCI	75.5±38.1	54.4±44.9	36.6±32.7	27.3±26.2	53.9±41.8	<0.001
AD	28 (40.6%)	22 (33.8%)	32 (69.6%)	3 (11.1%)	85 (41.1%)	<0.001
